# Serum Lactate Dehydrogenase Levels Reflect the Lung Injury Extension in COVID‐19 Patients at Hospital Admission

**DOI:** 10.1002/iid3.70168

**Published:** 2025-03-12

**Authors:** Maria Sofia Bertilacchi, Giulia Vannucci, Rebecca Piccarducci, Lorenzo Germelli, Chiara Giacomelli, Chiara Romei, Brian Bartholmai, Greta Barbieri, Claudia Martini, Michela Baccini

**Affiliations:** ^1^ Department of Pharmacy University of Pisa Pisa Italy; ^2^ Department of Electrical and Information Technology DIETI University of Naples Federico II Napoli Italy; ^3^ Department of Radiology Pisa University Hospital Pisa Italy; ^4^ Division of Radiology, Mayo Clinic Rochester Rochester Minnesota USA; ^5^ Department of Emergency Medicine Department Pisa University Hospital Pisa Italy; ^6^ Department of Statistics Computer Science, Applications University of Florence Florence Italy

**Keywords:** blood parameters, CALIPER ILD, chest computed tomography, COVID‐19, lactate dehydrogenase, lung injury

## Abstract

**Background:**

Several hematological and biochemical parameters have been related to the COVID‐19 infection severity and outcomes. However, less is known about clinical indicators reflecting lung involvement of COVID‐19 patients at hospital admission. Computed tomography (CT) represents an established imaging tool for the detection of lung injury, and the quantitative analysis software CALIPER has been used to assess lung involvement in COVID‐19 patients. Herein, the relationship between the lung involvement expressed by CALIPER interstitial lung disease (ILD) percentage and a set of blood parameters related to tissue oxygenation and damage in COVID‐19 patients at hospital admission was evaluated.

**Methods:**

We performed a retrospective and a prospective study involving 321 and 75, respectively, COVID‐19‐positive patients recruited from Pisa University Hospital. The association between CALIPER ILD percentages and selected blood parameters was investigated by a regression tree approach, after multiple imputations of the dataset missing values.

**Results:**

High serum lactate dehydrogenase (LDH) values appeared to be predictive of high CALIPER ILD percentages at hospital admission in both retrospective and prospective datasets, even if the predictive performance of the algorithm was not optimal.

**Conclusions:**

LDH levels could be evaluated as a tool for early identification of COVID‐19 patients at risk of extensive lung injury, as well as in fast screening procedures before hospitalization.

## Graphical abstract

The relationship between the lung involvement expressed by quantitative computed tomography (CALIPER) and a set of blood parameters related to tissue oxygenation and damage in COVID‐19 patients at hospital admission was evaluated in a retrospective and a prospective study involving 321 and 75, respectively, COVID‐19‐positive patients. The results evidence that the lactate dehydrogenase (LDH) serum levels could be considered as a tool for early identification of COVID‐19 patients at risk of extensive lung injury, as well as in fast screening procedures before hospitalization.

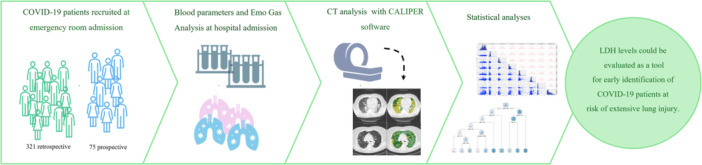

## 1 Introduction

Starting from December 2019, the world has experienced the spread of a new virus called Severe Acute Respiratory Syndrome Coronavirus 2 (SARS‐CoV‐2) and the related disease called COVID‐19 [1, 2]. SARS‐CoV‐2 virus has infected more than 650 million people with over 6.5 million deaths, representing one of the major global public health concerns [3, 4]. Individual responses to SARS‐CoV‐2 infection vary dramatically, ranging from asymptomatic or mild flu‐like symptoms to severe symptoms, including acute respiratory distress syndrome (ARDS) and death [5].

SARS‐CoV‐2, as well as other respiratory infections, is characterized by pulmonary injury [6]. A higher lung involvement is associated with higher functional impairment and poor quality of life, since it could lead to ARDS and intensive unit care admission [7]. Moreover, COVID‐19 pneumonia has been demonstrated to be associated with pulmonary sequelae [8]. The combination of clinical examination, imaging investigation, and blood parameters represents a source to predict the disease severity. However, the relationships among these aspects, especially during the first phase of the infection, remain difficult to undertake.

According to the World Health Organization, chest computed tomography (CT) represents one of the major imaging instruments for the detection of lung injury in COVID‐19 patients [9]. Typical early pulmonary CT images of COVID‐19 pneumonia show pulmonary parenchyma involvement revealing ground‐glass opacities. The injury becomes more extended and evolves in consolidation 7–10 days after the onset of the initial symptoms [10, 11]. Moreover, lung involvement and its visual assessment on CT have been used as a prognostic tool, for instance, to predict mortality in COVID‐19 intensive unit care patients [12]. The limit of the visual assessment, mainly due to the inter‐reader variability in observation [13], has been recently overcome by several automatic software such as Computer‐Aided Lung Informatics for Pathology Evaluation and Rating (CALIPER), used for the analysis and quantification of parenchymal lung abnormalities on chest CT. CALIPER has already been widely applied for the analysis and quantification of diffuse parenchymal lung abnormalities, measuring the distribution of ground‐glass opacity areas defined as the CALIPER interstitial lung disease (ILD) parameter [14]. Interestingly, recent studies highlight a significant increase in the CALIPER ILD parameter among COVID‐19 patients with poorer outcomes [15], as well as a rise in the CALIPER vascular‐related structure volume (VRS) percentage in patients admitted to the intensive care unit [16]. Moreover, CALIPER software has been used to quantify COVID‐19 pneumonia abnormalities in survivors from the acute phase to 24‐month follow‐up [17]. Unfortunately, the limited diffusion and high cost of CT automatic software CALIPER often restrict its use in patients' routine analysis. On the contrary, hematological assays are commonly employed. Despite some blood peripheral parameters that have been related to the pathogenesis and severity of COVID‐19 [18, 19, 20], the evidence about discriminant values able to reveal the entity of lung injury at the time of hospital admission is still limited [21]. Notably, identifying blood markers that correlate with the extent of lung damage could assist clinicians in more efficient and faster prediction of disease progression. This approach may reduce reliance on CT scans, lower healthcare costs, and help limit the spread of the virus.

Herein, the relationship between lung damage, quantified by CALIPER, and levels of specific blood parameters related to tissue oxygenation (hemoglobin [Hb], ferritin, partial arterial oxygen pressure [pO_2_], partial arterial carbon dioxide pressure [pCO_2_], and lactates) and tissue damage (lactate dehydrogenase [LDH]) was investigated using a retro‐prospective dataset from SARS‐CoV‐2‐positive patients at hospital admission. Separate analyses were conducted on the retrospective and prospective datasets, employing Classification and Regression Trees (CART). Due to a significant number of missing values in the prospective dataset, a preliminary multiple imputation procedure was applied.

Overall, the study aims to identify blood parameters that can early reflect lung damage in alignment with CT scan findings regardless of the specific mechanisms of lung injury. This could improve patient management during hospitalization by enabling clinicians to perform a rapid initial clinical assessment, which can later be complemented by CT scans.

## 2 Materials and Methods

### 2.1 COVID‐19 Patients: Recruitment and Inclusion Criteria

Two datasets were available reporting similar information on two sets of patients. The first dataset was retrospectively collected. It included COVID‐19 patients admitted to Pisa University Hospital with COVID‐19 symptoms from March to April 2020. Patients were included in the study if they had a SARS‐CoV‐2‐positive nasopharyngeal swab, analyzed by real‐time PCR, and confirmation of pulmonary involvement by chest CT.

The second dataset was prospectively collected. It included COVID‐19 patients admitted with symptoms to the same unit from May 2021 to September 2022. Only patients with a positive swab and positive chest CT were recruited.

The study procedures were approved by the local Ethical Committee (Protocol number 17368, Pisa, 14/05/2020 for the retrospective study; Protocol number 19275, 25/02/2021 for the prospective study) and the Great North West Area of Tuscany and were in accordance with the provisions of the Declaration of Helsinki. All participants gave written informed consent for the use of their clinical data and blood samples for research purposes.

### 2.2 Chest CT Analysis and Quantification of Interstitial Lung Abnormalities

The chest CT analysis was performed at the emergency room entry. All CTs were anonymized and analyzed with the automatic texture analysis software CALIPER, which performs a segmentation of right and left lungs and then a texture analysis of interstitial lung abnormalities. The CALIPER ILD parameter is derived by the sum of the percentage of ground‐glass and reticulation areas [22]. CT scans with severe motion artifacts or not technically adequate CALIPER CT segmentations were excluded.

### 2.3 Collection of Whole Blood and Isolation of Red Blood Cells and Plasma

Blood samples were collected at the hospital admission for each COVID‐19‐positive patient enrolled in the studies. The plasma and red blood cells were then isolated at the “Unità Operativa Biobanche” of the AOUP. Routine and nonroutine blood clinical analysis were performed at hospital admission before other treatments. Specifically, Hb was evaluated in whole blood, while ferritin and LDH concentrations were evaluated in the serum. PaO_2_, PaCO_2_, pH, lactates, and bicarbonates were assessed by arterial blood gas analysis. The main patients' features are summarized in Tables 1 and 2.

**Table 1:** Demographic and clinical data for the retrospective dataset. F, female; CV pathology, cardiovascular pathology; COPD, chronic obstructive pulmonary disease; HCO_3_^−^, bicarbonates. Data are presented as number or mean (%).

Demographic and clinical data (retrospective dataset)	
Demographic data	Mean
Age	67.40
	Count (%)
Sex (F)	104 (32)
Clinical data	
Smokers	18 (6)
Ex‐smokers	42 (13)
Hypertension	144 (46)
CV pathology	105 (33)
Asthma	19 (6)
COPD	37 (12)
Cerebrovascular pathology	32 (10)
Dementia	23 (7)
Solid cancer	43 (14)
Hematologic cancer	8 (3)
Hypercholesterolemia	53 (17)
Diabetes mellitus	61 (19)
Symptoms	
Fever	161 (51)
Cough	26 (54)
Shortness of breath	148 (47)
Diarrhea	58 (17)
Myalgia	42 (13)
Fatigue	47 (15)
	Mean (%)
Emogas analysis	
pH	7.4
Bicarbonates (HCO_3_^−^) (mEq/L)	24.7

**Table 2:** Demographic and clinical data for the prospective dataset. F, female; CV pathology, cardiovascular pathology; COPD, chronic obstructive pulmonary disease; HCO_3_^−^, bicarbonates. Data are presented as number or mean (%).

Demographic and clinical data (prospective dataset)	
Demographic data	Mean
Age	59
	Count (%)
Sex (F)	31 (41)
Clinical data	
Smokers	3 (4)
Ex‐smokers	19 (26)
Hypertension	39 (51)
CV pathology	29 (40)
Asthma	8 (11)
COPD	13 (17)
Cerebrovascular pathology	11 (15)
Solid cancer	12 (16)
Hematologic cancer	11 (14)
Dyslipidemia	17 (21)
Symptoms	
Fever	43 (59)
Cough	26 (35)
Dyspnea	37 (49)
Asthenia	12 (16)
	Mean (%)
Emogas analysis	
pH	7.5
Bicarbonates (HCO_3_^−^) (mEq/L)	25.6

Only for the prospective dataset, the quantification of angiotensin‐converting enzyme 2 (MBS2506383‐96), angiotensin 1–7 (Cod. MBS084052‐96), and hypoxia‐inducible factor 1α (Cod. MBS282197‐96) in plasma sample, as well as 2,3‐biphosphoglycerate (Cod. MBS288269‐96) in red blood cells, was performed using commercial enzyme‐linked immunosorbent assay (ELISA) kits.

### 2.4 Descriptive Analysis

Separate descriptive analyses were performed for the retrospective and prospective datasets. The variables were summarized in terms of minimum value, maximum value, mean, median, and interquartile range. For each variable, the count and the percentage of missing values were also reported. The marginal association between each pair of variables was evaluated by calculating the Spearman's correlation coefficient (*r* = 0.00–0.19 indicates a very weak correlation, *r* = 0.20–0.39 indicates a weak correlation, *r* = 0.40–0.59 indicates a moderate correlation, *r* = 0.60–0.79 indicates a strong correlation, and *r* = 0.80–1.00 indicates a very strong correlation [23]), with its 90% confidence intervals (CIs).

### 2.5 Tree‐Based Analysis

CALIPER ILD was considered as the outcome for the following subset of features: age, Hb, Ferritin, LDH, pO_2_, pCO_2_, and lactate. The association between the outcome and the selected features was analyzed by specifying a regression tree (RT) [24]. RTs are a class of nonparametric predictive models that lead to a piecewise constant representation of the regression function by partitioning the predictor space. Details on CART algorithm and RTs theory can be found in [24, 25, 26]. The analysis of the retrospective dataset was restricted to the units with non‐missing outcomes (*n* = 267), while we handled missing predictors following a standard procedure commonly implemented within the CART algorithm [21, 22].

To express the relative contribution of each explanatory variable in predicting the outcome, we calculated the so‐called variable importance (VI), defined as in [24]. The VI values were scaled to sum to 100.

Regarding the prospective dataset, the analysis was performed on all the units (*n* = 75), including those with missing outcomes. In fact, the first step of the analysis consisted of a multiple imputation of missing values. Specifically, under the Missing At Random assumption, we performed a Multiple Imputation by Chained Equations (MICE) [27], using random forests as predictive models [28]. For the multiple imputation, all the clinical and laboratory information collected on the 75 patients (for a total of 31 variables) was considered. 20 imputed datasets were obtained, on which we conducted the same tree‐based analysis performed on the retrospective dataset. The results obtained on the 20 imputed datasets were finally combined in an overall result [28].

### 2.6 Cross‐Validation Scheme

**Figure 1:**
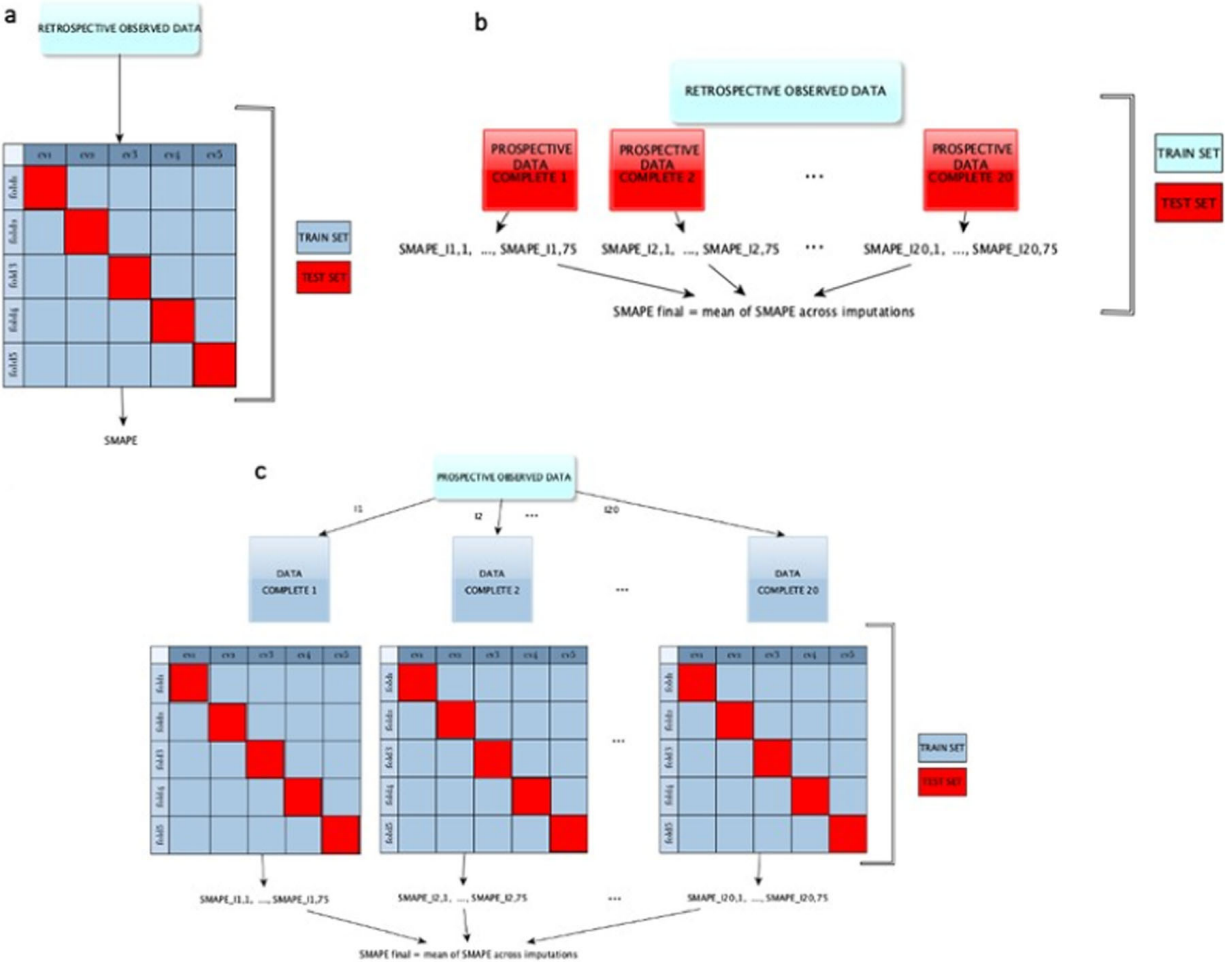
Cross‐validation scheme: (a) fivefold cross‐validation adopted in the retrospective dataset analysis; (b) out‐of‐sample validation of the model developed on the retrospective dataset, performed on the multiply imputed prospective datasets; (c) fivefold cross‐validation, combined with multiple imputation, adopted in the prospective dataset analysis.

The predictive performance of the RT models was evaluated via Cross Validation, for both the retrospective and the prospective datasets (Figure 1).

In the case of the retrospective dataset, a fivefold Cross Validation was employed and the sMAPE (symmetric mean absolute percentage error) was computed as a measure of discrepancy between observed and predicted values of the outcome [29, 30].

In the case of the prospective dataset, the fivefold Cross Validation was integrated with the MI. Specifically, separate fivefold Cross Validations were performed on the 20 imputed datasets and a final performance measure was obtained by averaging over the 20 sMAPE. The same fold definition was used for all the Cross Validations conducted on the 20 imputed datasets.

We also assessed the out‐of‐sample predictive performance of the model developed on the retrospective data by using as test sets the 20 imputed datasets arising from the multiple imputations on the prospective dataset.

### 2.7 Code Availability

All the analyses have been performed with R software, version 4.0.2. The code, written by the authors, involved the R packages rpart for RTs and mice for multiple imputations. It is available upon request to the authors. All figures have been created with R software, version 4.0.2, with the exception of Figure 1, which has been created with yEd version 3.22.

## 3 Results

### 3.1 Demographic and Clinical Data

The retrospective study enrolled 321 patients, 104 (32%) women and 217 (68%) men, with a mean age of 67.4 years (range: 96–24). Among them, 18 (6%) were smokers, 42 (13%) were ex‐smokers, and 229 (71%) had never smoked. The most common comorbidities of the analyzed cohort were hypertension (46%), followed by cardiovascular diseases (33%), diabetes mellitus (19%), hypercholesterolemia (17%), cancer (14%), and cerebrovascular diseases (10%). Typical symptoms were cough (54%), fever (51%), shortness of breath (47%), diarrhea (17%), fatigue (15%), and myalgia (13%).

The prospective study enrolled 75 COVID‐19 patients, 31 (41%) women and 44 (59%) men, with a mean age of 59 years (range: 27–89). Among them, 19 (26%) were ex‐smokers, 51 (69%) had never smoked, and only 3 (4%) patients were smokers. The most common comorbidities were hypertension (51%), followed by cardiovascular risk (40%), dyslipidemia (21%), and solid and blood tumors (16% and 14%, respectively). At the hospital's admission, the most common symptoms were fever (59%), dyspnea (49%), cough (35%), and asthenia (16%).

Among these 75 COVID‐19 patients, 20 (27%) required intensive unit care or sub‐intensive unit care hospitalization, while the other 55 (73%) patients were admitted to non‐intensive unit care. Finally, 5 (7%) patients had thrombotic complications, while 26 (35%) had bacterial overinfections. Tables 1 and 2 contain all the demographic, clinical, symptoms, and emogas analysis data of retrospective and prospective datasets, respectively.

### 3.2 Analysis of Retrospective Data

**Figure 2:**
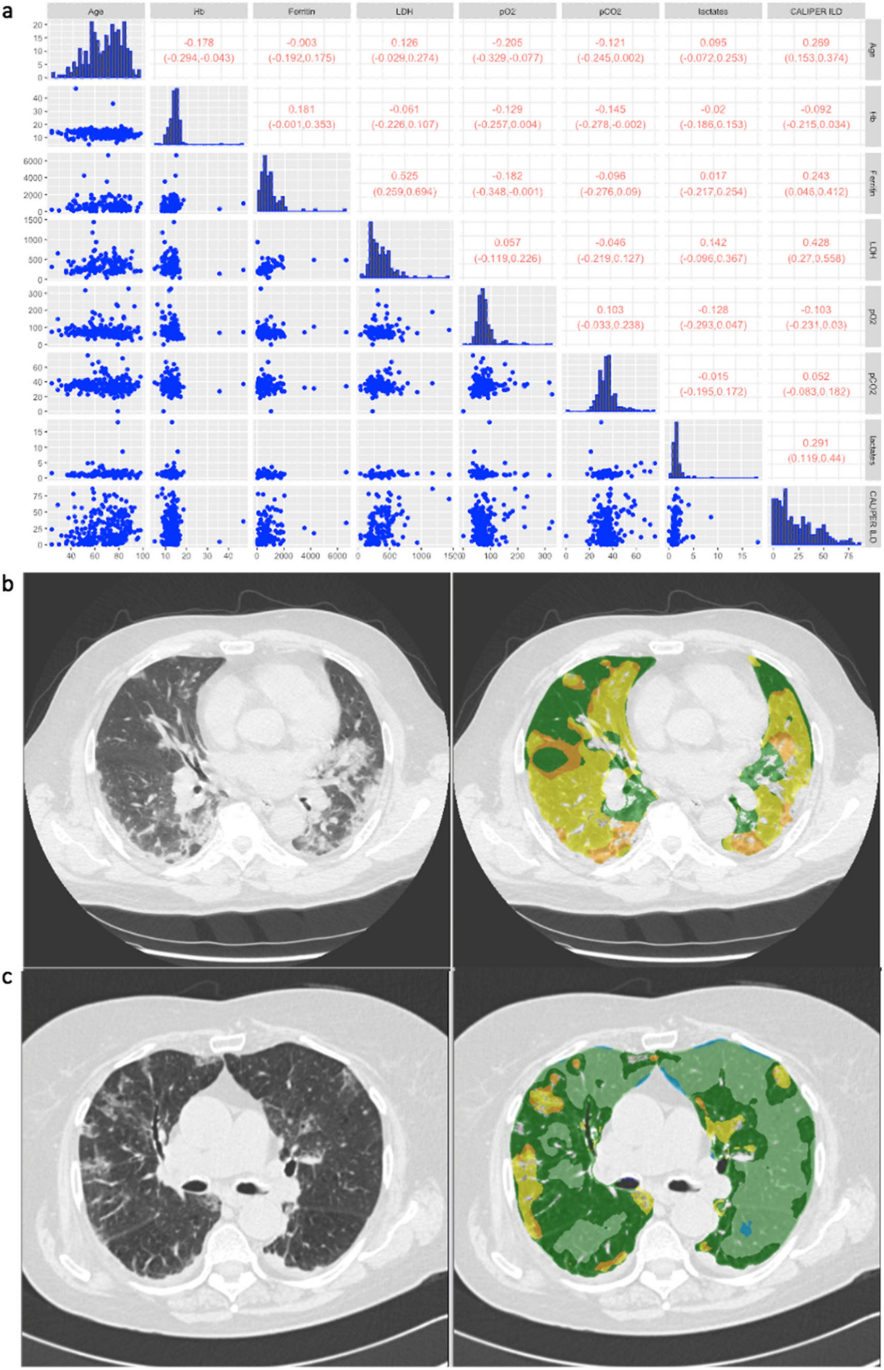
(a) Scatterplot matrix and correlation among variables for the retrospective dataset. Histograms of the variables are reported in the diagonal, bivariate scatterplots are reported in the lower panel, and Spearman's correlation coefficients with their 90% bootstrap confidence intervals (90% CIs) are reported in the upper panel. (b) Representative lung CT images and CALIPER segmentation images in a patient showing higher pulmonary impairment as indicated by the expansion of ground‐glass opacity, typical COVID‐19 lung abnormalities. (c) Representative lung CT images and CALIPER segmentation images in a patient showing lower pulmonary impairment as indicated by the expansion of ground‐glass opacity, typical COVID‐19 lung abnormalities. Different color schemes are used to underline different damaged lung areas: light and dark green represent the normal lung areas, yellow the ground‐glass opacity, and orange the reticular pattern.

The summary statistics for the selected variables and the outcome CALIPER ILD in the retrospective dataset are reported in Table 3. Patients with missing outcomes (*n* = 54, 16.82%) were excluded from this analysis. The scatterplot data matrix of the observed data is reported in Figure 2a: the histograms of the variables are reported in the diagonal, the bivariate scatterplots in the lower panel, and the Spearman's correlation coefficients with their 90% bootstrap CIs (90% CIs) in the upper panel. Most of the variables have nonnormal and skewed distributions. The bivariate scatterplots do not show specific behavior or trends, confirming small/moderate correlations between variables. The highest correlation for the outcome CALIPER ILD was calculated for the variable LDH (0.428; 90% CI: 0.27–0.558). Notably, patients with higher serum LDH levels also show a major pulmonary impairment, as indicated by ground‐glass opacities (an example is reported in Figure 2b) when compared to patients with lower LDH levels, which showed lower lung damage (Figure 2c).

**Table 3:** Descriptive analysis for the retrospective dataset. Descriptive statistics (minimum, first quartile Q1, median, mean, third quartile Q3, maximum, number of missing values NA, and the percentage of missing values) for the outcome (CALIPER ILD) and the selected variables (age, lactates, pO_2_, pCO_2_, Hb, ferritin, and LDH), *n* = 267.

	Min	Q1	Median	Mean	Q3	Max	NA	% Missing
Age	24	57	69	67.40	79	98	—	—
Lactates (mmol/L)	0	0.90	1.10	1.43	1.50	18.20	130	48
pO_2_ (mmHg)	0	58	71	77.21	83	326	24	8
pCO_2_ (mmHg)	0	30	34	35.10	38	76	31	11
Hb (g/dL)	4.88	12.10	13.50	13.40	14.70	47.10	6	2
Ferritin (µg/L)	34	346.80	636	817.70	980.20	6657	147	55
LDH (U/L)	37	216	313	351.2	446	1439	116	43
CALIPER ILD	0.05	7.17	18.21	23.49	35.71	85.68	—	—

**Figure 3:**
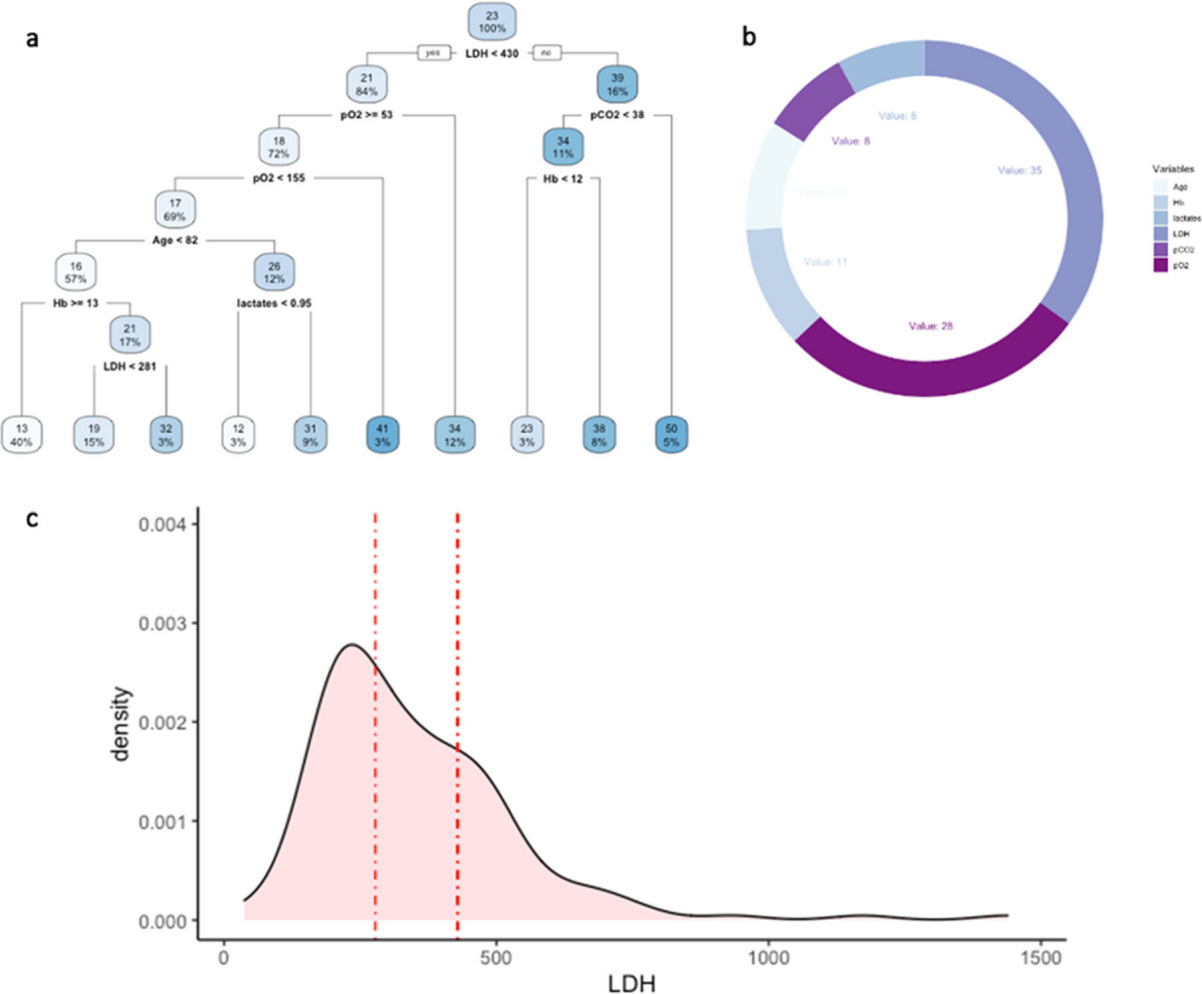
Analysis of the retrospective dataset. (a) Regression tree for the variable CALIPER ILD. Each node reports the average value of the outcome and the percentage of units in the partition. (b) Variable importance of the explanatory variables from the regression tree. (c) Distribution of the variable LDH (U/L) in the retrospective dataset and splitting points (dashed red lines) for the same variable arising from the analyses on the training sets defined within the fivefold CV.

Thus, to evaluate the prediction capacity of serum variables on the outcome variable “CALIPER ILD,” an RT was estimated on the retrospective data and depicted in Figure 3a. At the root node, all 267 units were included, and the average value of CALIPER ILD was 23%. The first split was defined by the condition “LDH < 430”: the average CALIPER ILD for the 224 patients (84%) who meet this condition was 21% and 39% for the others. Going down the tree, the units for which the condition “LDH 430” was verified were in turn split according to the condition “pO_2_ ≥ 53”: if “pO_2_ ≥ 53” (192 patients, 72%), the average CALIPER ILD was 18%; if “pO_2_ < 53” (32 patients, 12%), the average was 34%, and so on. The average value of the outcome and the final partition are reported in the terminal nodes.

The variable importance was calculated to evaluate the predictive capacity of each explanatory variable used in the tree model (Figure 3b). The values are scaled to sum to 100, and the ones lower than 1% are not shown [23]. The highest VI was calculated for the variable LDH (35), followed by pO_2_ (28).

The predictive performance of the tree, evaluated through the fivefold Cross Validation, was quite low, with a sMAPE of 77% (upper bound 200%). Figure 3c shows the distribution of variable LDH in the dataset as well as the splitting points (represented by the dashed lines) in the 5 RTs estimated on the training sets during CV (only the first split was considered in the case of more than one split involving LDH). In four trees, the splitting point for LDH was close to 430; in the remaining tree was 277.5.

The out‐of‐sample predictive performance estimated by using the retrospective data as the training set and each multiple imputed prospective dataset as the test set was quite low, with an average sMAPE of 66%.

### 3.3 Analysis of Prospective Data

**Figure 4:**
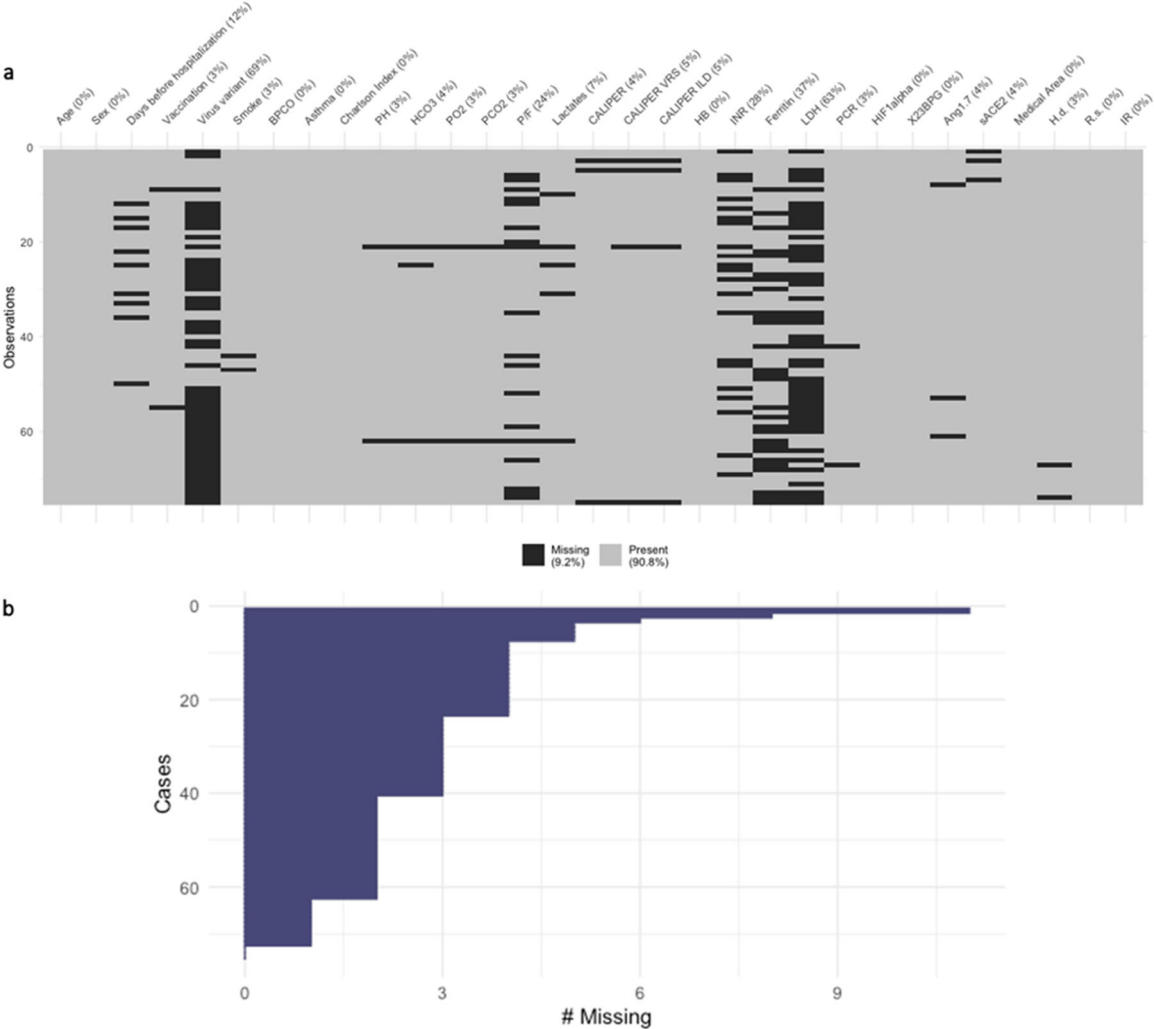
Pattern of missing values in the prospective dataset. (a) Matrix reporting the pattern of missing values by subject (row) and variable (column), in the dataset on which multiple imputation was performed. (b) Bar diagram reporting for each patient the number of missing values in the variables used in the regression tree analysis (from the subject with the highest number of missing (#11) to the subject with the lowest one).

The pattern of the missing data per variable, the percentage of total missing (in the legend), and the percentage of missing per variable (in the column labels) for the prospective dataset are shown in Figure 4a. Figure 4b shows the number of missing per subject (from the subject with the highest number of missing (#11) to subject with the lowest). Therefore, a Multiple Imputation procedure was performed, resulting in 20 complete datasets.

**Figure 5:**
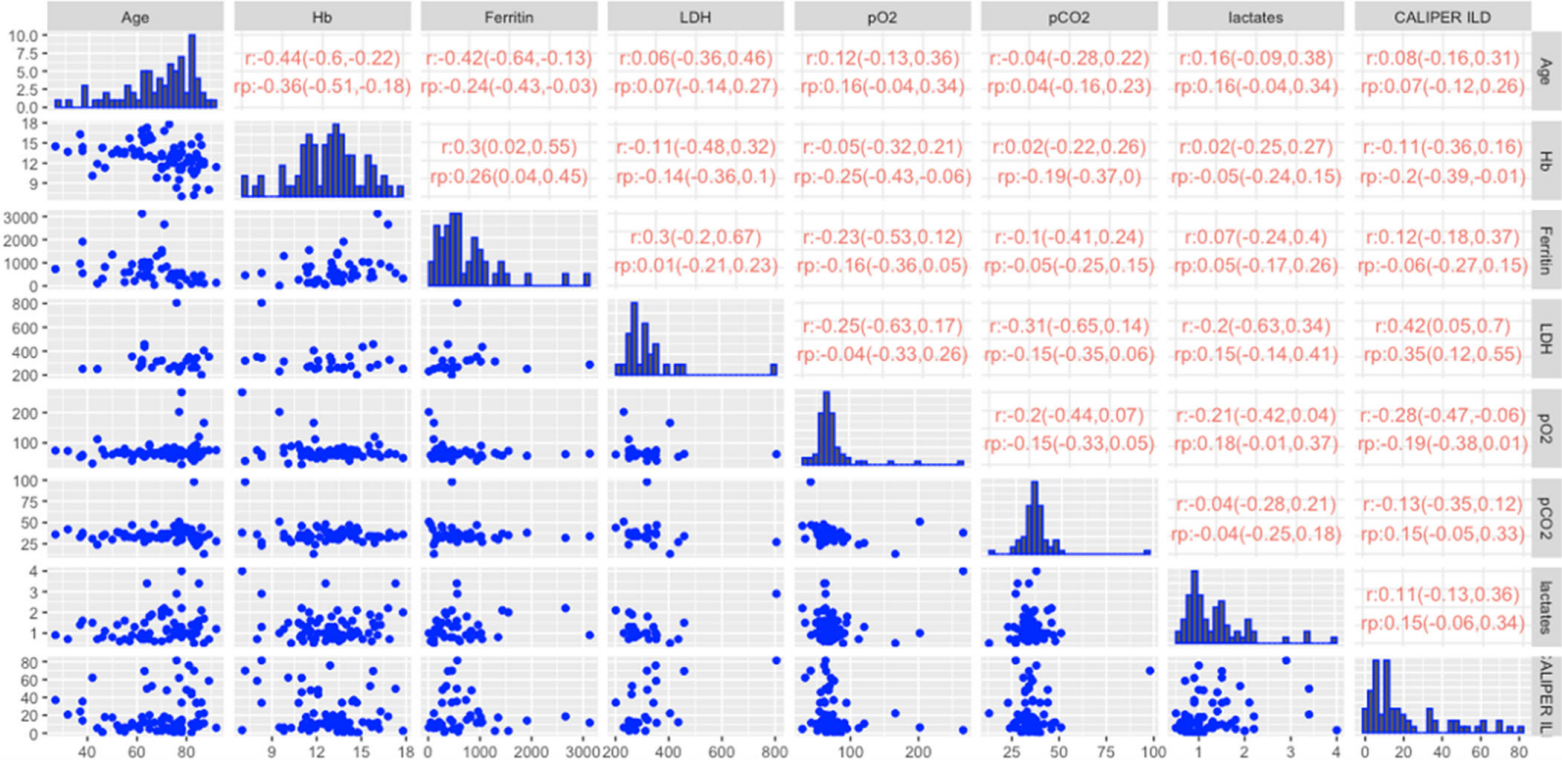
Scatterplot matrix and correlation between variables of the prospective dataset. Histograms of the variables (diagonal), bivariate scatterplots (lower panel), and Spearman's correlation coefficients (*r*) calculated on the subset of complete cases and on the pooled ones (*r_p_*), with their 90% confidence intervals (upper panel).

In Table 4, the summary statistics for the selected variables and the outcome in the prospective dataset are reported. Among the variables with the highest number of missing values were LDH and ferritin. Figure 5 shows the scatterplot data matrix. Most of the variables have nonnormal and skewed distributions. The bivariate scatterplots do not show specific behavior or trends, and the correlations between variables are small/moderate. Notice that we reported the Spearman correlation coefficients (*r*) calculated on the subset of complete cases and the pooled ones ($r_{p}$) calculated over the 20 imputed datasets. The highest marginal association with the outcome was estimated for the variable LDH: r=0.42 (CI 0.05; 0.7) and $r_{p}=0.35$ (CI 0.12; 0.55).

**Table 4:** Descriptive analysis for the prospective dataset. Descriptive statistics (minimum, first quartile Q1, median, mean, third quartile Q3, maximum, number of missing values NA, and the percentage of missing values) for the outcome (CALIPER ILD) and the selected variables (age, lactates, pO_2_, pCO_2_, Hb, ferritin, and LDH), *n* = 75.

	Min	Q1	Median	Mean	Q3	Max	NA	% Missing
Age	27	62	73	69.03	80.50	92	—	—
Lactates (mmol/L)	0.50	0.80	1.10	1.29	1.50	4	5	6
pO_2_ (mmHg)	29	61	67	73.42	75	266	2	2
pCO_2_ (mmHg)	13	32	35	35.82	38	98	2	2
Hb (g/dL)	7	11.45	13	12.87	14.30	17.80	—	—
Ferritin (µg/L)	12	274.50	551	696.50	886.50	3127	28	37
LDH (U/L)	201	261.80	299.50	321.80	345	805	47	62
CALIPER ILD (%)	0.53	6	12.16	20.85	33.97	81.36	4	5

**Figure 6:**
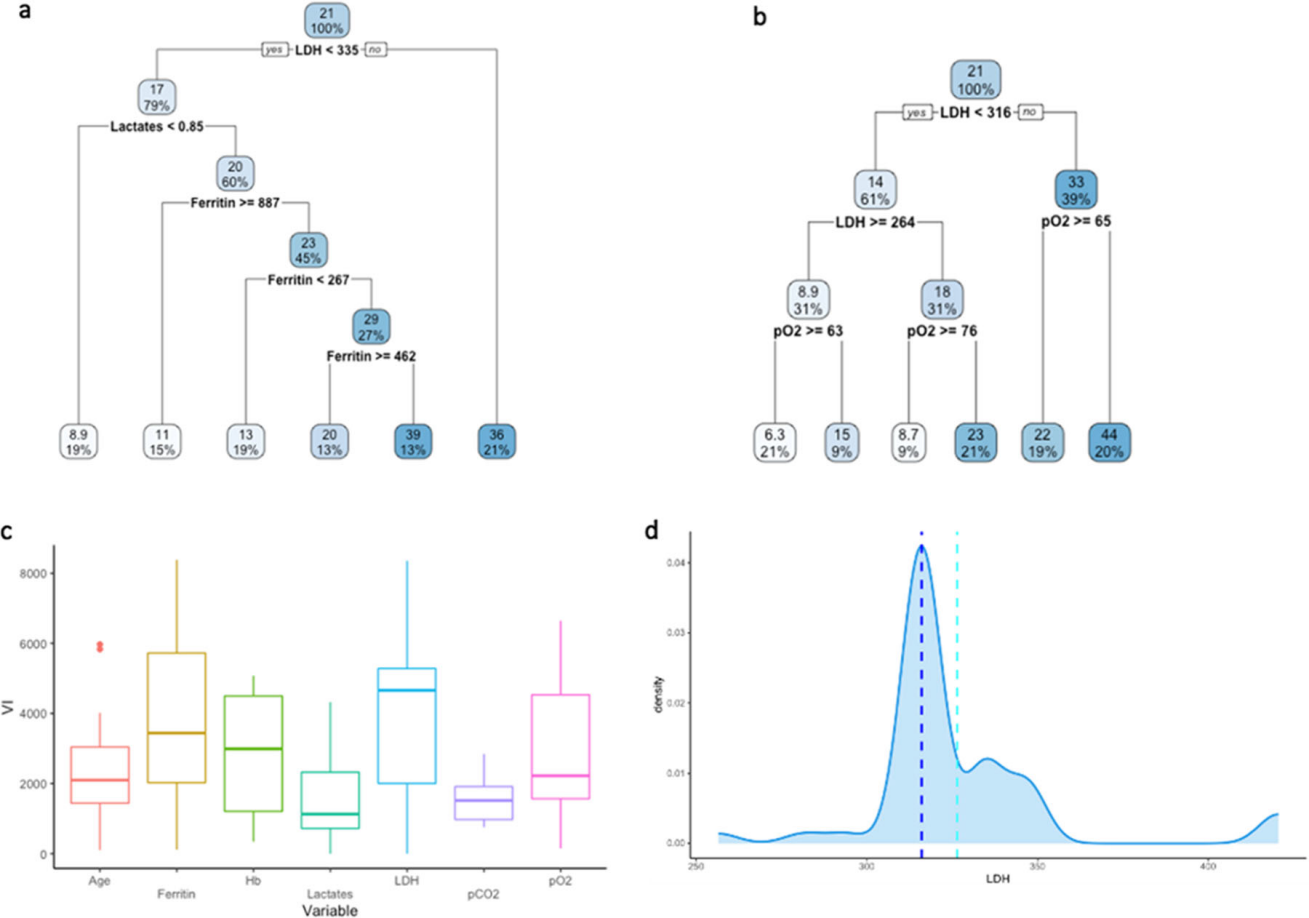
Analysis of the prospective dataset. Regression trees estimated for the variable CALIPER ILD on the first (a) and the second (b) imputed datasets. Each node reports the average value of the outcome and the percentage of units in the partition. (c) Box plots of the variable importance values for age, ferritin, Hb, lactates, LDH, pCO_2_, and pO_2_, arising from the regression trees obtained on the 20 imputed datasets. (d) Distribution of the variable LDH (U/L), vertical lines corresponding to mean (light blue dashed vertical line) and median (blue dashed vertical line) of the splitting points for the same variable arising from the analyses on the training sets defined within the fivefold CV.

For the prospective data, we estimated an RT separately for each of the 20 imputed datasets. As an example, the RTs on the first two imputed datasets are shown in Figure 6a,b (the remaining pictures of RTs can be found in Supporting Information, Figure S1). In both RTs, LDH was the first splitting variable, suggesting that LDH is an important predictor of the outcome. The importance of LDH was confirmed also by the VI values reported in Figure 6c, where the boxes show, for each variable, the distribution of the VI values arising from the analyses performed on the 20 imputed datasets. In Table S1, we also reported for each variable the imputation‐specific VI values, from Imputation 1 to Imputation 20. An average sMAPE of 76% was obtained from the fivefold CV.

Finally, the distribution of LDH in the prospective dataset is shown in Figure 6d together with the mean and median values for the splitting points (light blue and blue dashed vertical lines) arising from the RTs estimated over the training sets defined within the fivefold Cross Validation (5 training sets for each of the 20 imputed datasets). Only the first split was considered when more than one split involved LDH. The average value of the LDH splitting points was 326.41 (light blue dashed line), while the median value was 316 (blue dashed line).

## 4 Discussion

Herein, we reported the results of an analysis conducted on 321 patients (retrospective dataset) and 75 patients (prospective dataset) affected by COVID‐19, in which the lung damage was evaluated and correlated to blood parameters such as lactates, pO_2_, pCO_2_, Hb, ferritin, and LDH at the hospital admission. These blood parameters were chosen from a larger dataset since they could contribute to the definition of the severity and development of COVID‐19 disease [31]. Indeed, SARS‐CoV‐2 primarily attacks pulmonary tissues and impairs gas exchange leading to systemic hypoxia [32]. Therefore, parameters related to tissue oxygenation, such as Hb, ferritin, pO_2_, pCO_2_, and lactates, have been chosen. Moreover, systemic hypoxia leads to ARDS and lung damage over time. For this reason, LDH has been selected as a peripheral damage parameter [33].

From the descriptive analyses, a moderate marginal correlation arose between CALIPER ILD percentage and age, ferritin, LDH, and lactate levels in the retrospective dataset. On the other hand, in the prospective dataset, a certain association was only observed between CALIPER ILD and LDH levels. In both datasets, LDH was positively correlated with the ferritin level.

To deeply investigate whether the selected blood parameters were able to predict the CALIPER ILD percentage, a more detailed analysis was performed, based on RTs. The RT models, considering the contribution of all explanatory variables at the same time, confirmed LDH as a possible predictor of CALIPER ILD percentage. Interestingly, our analysis also provided insights about a possible discriminant threshold for the LDH values. Indeed, the splitting value for LDH in the RTs was always over the median value and especially over the physiological LDH range values (135–215 U/L). Therefore, our results seem to indicate that an over‐threshold serum LDH value higher than the physiological one at hospital admission could predict higher CALIPER ILD percentages. Other studies have shown that LDH levels are higher in COVID‐19 patients and tried to provide a prognostic value of LDH for COVID‐19 severity [34, 35, 36], in accordance with our analysis; however, our work sheds light on a specific direct connection focusing on a specific lung damage indicator.

Of note, LDH is a constitutive protein expressed in all living cells and is widely distributed in almost all tissues, with the highest expression found in the heart, kidney, liver, and blood cells, whereas lesser amounts are found in the lung, smooth muscles, and brain [37]. Physiologically, LDH activity is essential during hypoxic or anaerobic cellular state, catalyzing the reversible conversion of pyruvate into lactate with the concomitant oxidation of nicotinamide adenine dinucleotide [37, 38, 39]. Interestingly, no strong correlation or correlation between CALIPER ILD and lactate levels was found in retrospective and prospective studies, suggesting that serum LDH is a marker of cellular breakdown rather than a marker of a hypoxic state.

LDH is released in the extracellular environment as a consequence of cell death, thus representing a cell damage and lysis marker [40] in myocardial infarction [41] but also in different lung disorders such as several respiratory and interstitial disorders (ARDS and idiopathic pulmonary fibrosis) and lung obstructive disease [42, 43].

Moreover, serum LDH represents a biomarker for assessing the survival of patients with brain metastasis from small‐cell lung cancer [44], as well as a diagnostic and prognostic factor in patients with non‐small cell lung cancer [45, 46]. Particularly, the LDH serum levels provide additional information about pulmonary and lung endothelial injury.

Accordingly, during the acute phase of SARS‐CoV infection [47], an increase in total serum LDH levels has been observed as a result of massive tissue destruction. Especially during SARS‐CoV‐2 infection, LDH has already been demonstrated as a predictor of respiratory failure in patients, expressed by the damage index ratio PaO_2_/FiO_2_ [48] and as an independent factor for predicting severity and mortality [49, 50, 51]. An interplay between circulant serum LDH levels and the extent of lung involvement measured using semi‐quantitative CT‐analysis [52, 53] has been reported, in accordance with our results. However, these data are derived by semi‐quantitative scoring through a visual assessment of CT by radiologists with different experiences and are affected by high inter‐ and intra‐observer variability and time‐consuming [13]. To overcome these limits, quantitative automatic CT analysis has already been used to correlate lung CT involvement and serum LDH. The use of automatic CT densitometric analysis has underlined the correlation between LDH and the progression of COVID‐19 pneumonia, according to our results [54]. However, CALIPER CT automatic analysis represents a more specific technique with prognostic validity for the detection of interstitial disease. Particularly, the CT analysis reported in that study has been performed using an automatic densitometric tool that calculates the distribution of the pixels of the CT image, as a percentage of total lung volume, according to their density. Different thresholds were identified based on the density, and COVID‐19 pulmonary involvement has been attributed to the part of the lung that is denser than a healthy lung; of note, these density areas have been arbitrarily referred to as ground‐glass and reticulation. For this reason, that type of analysis is not able to discriminate the observed lung abnormalities from other causes of the increase in lung density not referable to ground‐glass or reticulation; in contrast, CALIPER software is a texture analysis based on an algorithm by which voxels (pixels 3D volume) are compared in pathological anatomy databases and allow the specific detection of interstitial lung abnormalities, specifically for COVID‐19 lung involvement to recognize ground‐glass and reticulation.

Notably, CALIPER software has been recently demonstrated to be able to allow quantification of lung abnormalities in the setting of acute COVID‐19 pneumonia and can be used to track their changes in extent and morphology over time [17]. Moreover, a higher percentage of ILD and vascular pulmonary‐related structure volume on chest CT quantified with CALIPER had been found in COVID‐19 patients with a worse disease outcome [15].

Definitely, our results show a quite clear relationship between serum LDH levels and lung involvement. Furthermore, the evaluated percentages of CALIPER ILD, correlating with LDH serum levels, suggest LDH as a potential indicator of severe lung disease extension at the time of hospital admission and a predictor of the disease severity. Additionally, we also found a positive correlation between LDH and serum ferritin levels, highlighting an inflammatory *scenario* in the COVID‐19 process beyond cell death. Ferritin is a shell protein able to sequester iron into its core. It plays a crucial role during infection since it is released from M1‐macrophage phenotype into circulation to reduce iron availability to pathogens [55]. Especially, it has been already described as an inflammatory marker during SARS‐CoV‐2 infection and, together with LDH, has been correlated with ARDS development in COVID‐19 patients [56].

Some limitations of the study must be considered. Regarding the statistical methods, we used an RT approach because RTs are simple to understand through their graphical representation and appealing as they easily deal with nonlinearity and interactions. In addition, RTs can easily deal with missing values in the predictors through surrogate variables. In our application, this feature allowed us to estimate the RT on the retrospective dataset by simply excluding units with missing outcomes (16.82%). Missing data imputation was instead necessary for the prospective dataset, because of the small sample size and the presence of missing values also in the response variable. It remains a limitation as these imputed values are estimates and may not fully reflect the actual measurements, potentially influencing the robustness of the findings. However, RTs are not without drawbacks. Typically, a limitation of the RT is the high variability of the trees: a small perturbation in the data can result in a very different series of splits. This could compromise the utility of the trees in terms of interpretation and/or prediction accuracy [57]. In our study, the quite low SMAPE arising from both the internal CV and the external validation suggests the need to also explore ensemble methods (i.e., random forests and boosting trees) that could provide more stable results, even if, in general, with moderately small samples as the ones in this study, simple methods should be preferred. Regarding the selected cohorts, an unforeseen selection bias should be considered for the retrospective dataset. Additionally, the study incorporates both retrospective and prospective datasets, encompassing patients from two distinct waves of infection (2020 for the retrospective dataset and 2021 for the prospective one), which could introduce variability due to differences in clinical management and disease progression between the waves.

## 5 Conclusion

In conclusion, our study suggests the utility of LDH as a peripheral parameter able to reflect the lung fraction involvement at the early stage of the disease. Together with the patient's symptoms, the quantification of serum LDH, which can be obtained through simple routine blood tests without requiring hospitalization, could be used by clinicians as an essential tool during fast screening procedures, to perform early identification of COVID‐19 patients at risk of extensive lung involvement and consequently faster hospitalization procedures. Notably, despite advanced imaging tools remaining a gold standard to assess lung involvement, several clinical setting lacks access to this software highlighting the relevance of identifying a peripheral marker such as serum LDH.

Future studies should aim to validate the utility of LDH as a predictive parameter for lung involvement in COVID‐19 patients by including larger and more diverse cohorts. Furthermore, multicenter studies are essential to confirm the reproducibility of these findings across different healthcare settings, particularly in resource‐limited areas where CT scans may not be readily available. Additionally, developing predictive models that integrate LDH levels with clinical symptoms and other routine tests could enhance early risk stratification and guide faster clinical decision‐making. This idea could be translated to wider studies considering a wider variety of diseases involving lung damage, thus defining the role of serum LDH in the lung disease spectrum.

## Author Contributions

**Maria Sofia Bertilacchi:** data curation, formal analysis, investigation, writing – original draft, writing – review and editing. **Giulia Vannucci:** data curation, formal analysis, investigation, writing – original draft, writing – review and editing. **Rebecca Piccarducci:** supervision, writing – review and editing. **Lorenzo Germelli:** supervision, writing – review and editing. **Chiara Giacomelli:** conceptualization, data curation, funding acquisition, supervision, writing – review and editing. **Chiara Romei:** conceptualization, funding acquisition, project administration, writing – review and editing. **Brian Bartholmai:** Methodology. **Greta Barbieri:** Data curation. **Claudia Martini:** conceptualization, funding acquisition, project administration, supervision, writing – review and editing. **Michela Baccini:** conceptualization, data curation, methodology, project administration, writing – review and editing.

## Conflicts of Interest

The authors declare no conflicts of interest.

## Supporting information

Supporting information.

Supporting information.

## Data Availability

The datasets used in the current study are available from the corresponding author. This article reports only a portion of the available data collected during this study.
