# Supplementary material for: Serum Lactate Dehydrogenase Levels Reflect the Lung Injury Extension in COVID‐19 Patients at Hospital Admission
Source: Immun Inflamm Dis. 2025 Mar 12;13(3):e70168. doi: 10.1002/iid3.70168 (PMC11898011; doi:10.1002/iid3.70168)
Supplement: Supplementary file 1 — Supporting information. [file IID3-13-e70168-s001.docx]

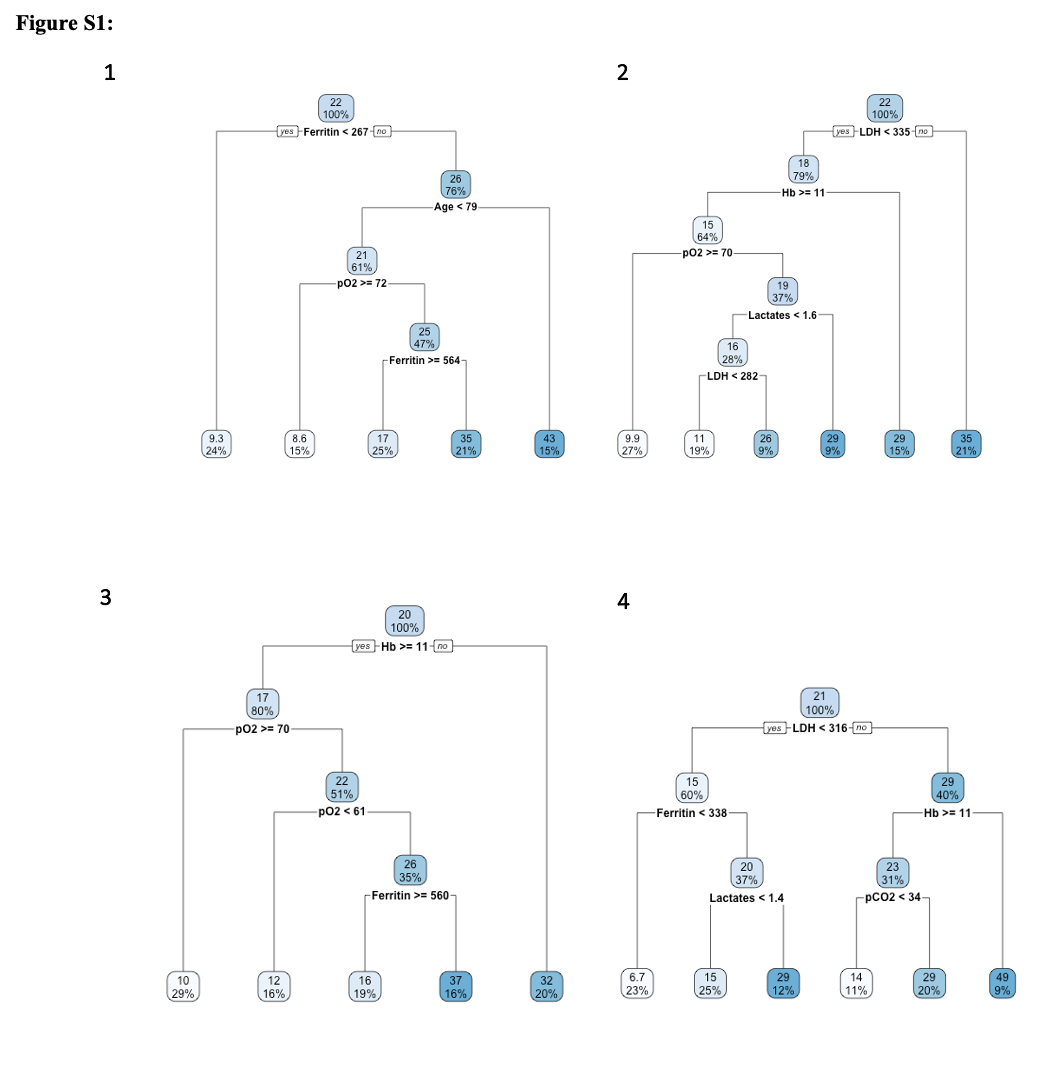


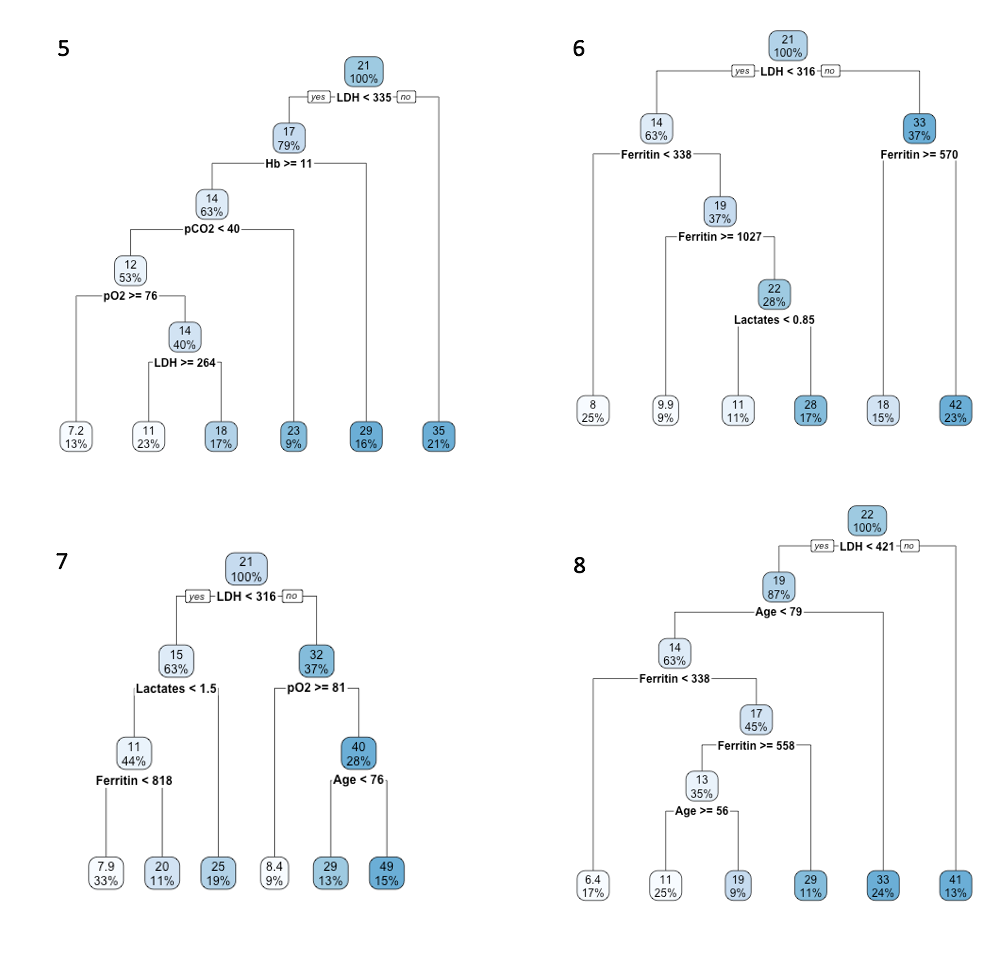

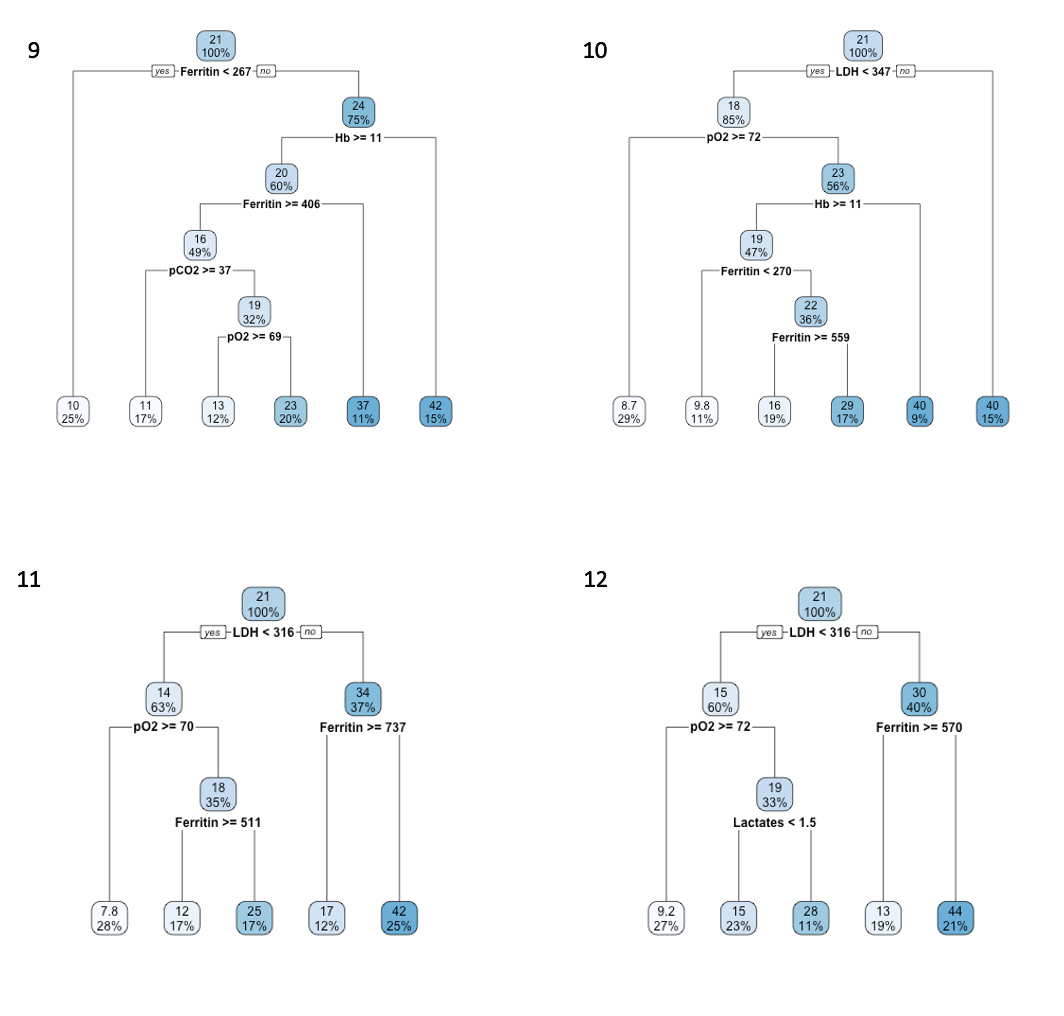

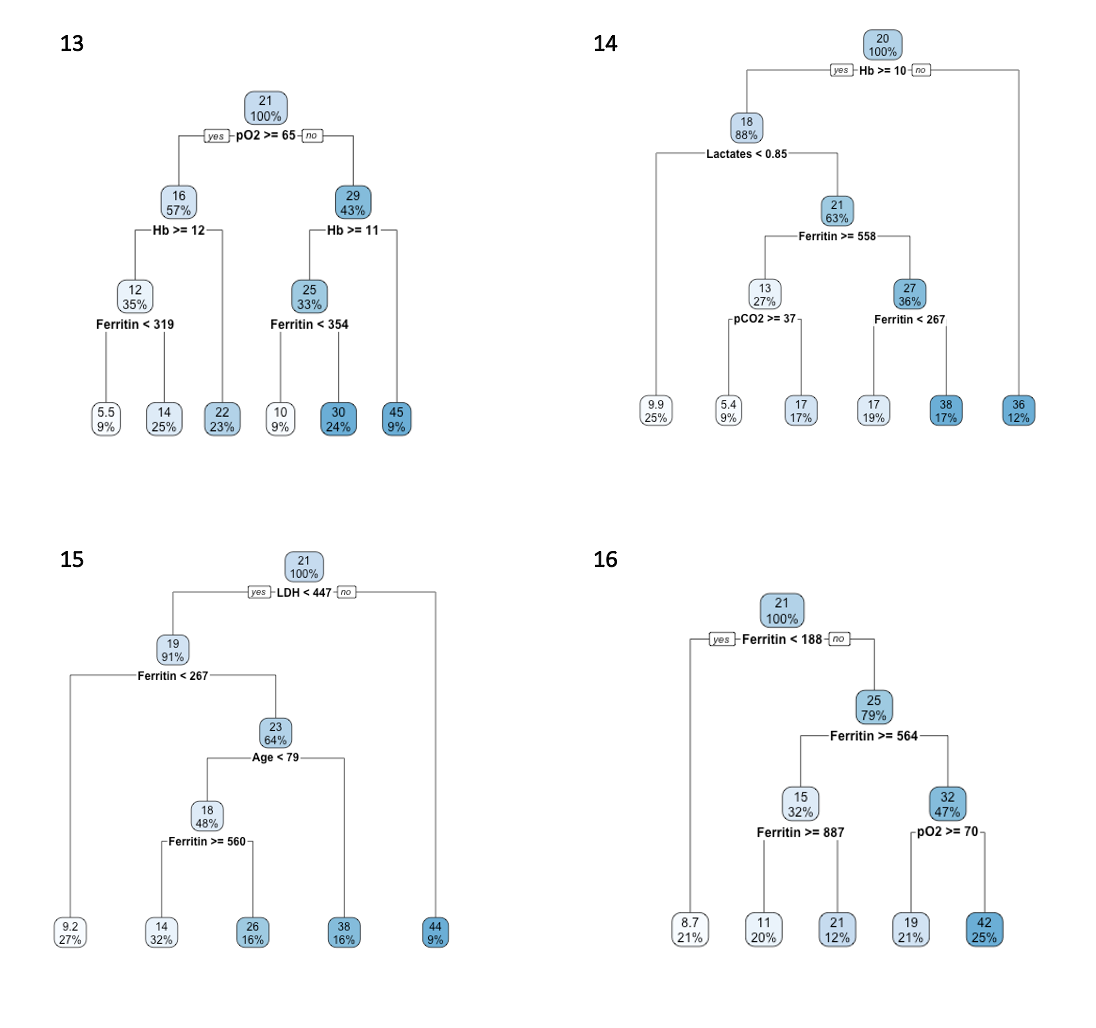

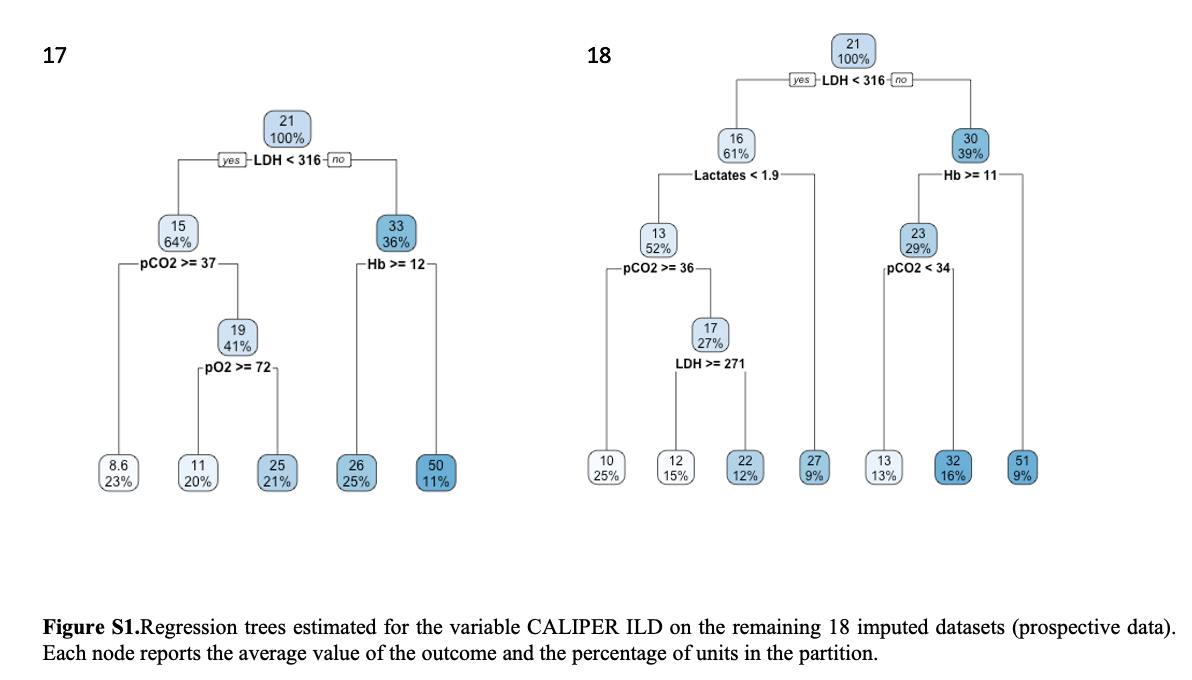


**Appendix:**

Optimised Study Group: Davide Bacciu, Andrea Barucci, Rita Mara Brizzi, Rossana Buongiorno, Laura Burzagli, Salvatore Massimiliano Cammisuli, Nicoletta Carpenè, Laura Carrozzi, Alessandro Celi, Daniele Chiappini, Davide Chimera, Sara Colantonio, Valentina Colcelli, Leonardo Colligiani, Gennaro D’Angelo, Chiara Del Carlo, Roberto Carpi, Annalisa De Liperi, Chiara Deri, Massimiliano Desideri, Francesco Faita, Marco Falcone, Salvatore Claudio Fanni, Paolo Ferragina, Maria Franzini, Luciano Gabbrielli, Luna Gargani, Danila Germanese, Lorenzo Ghiadoni, Simone Lapi, Chiara Marzi, Fabrizia Mealli, Emanuele Neri, Francesca Nocchi, Roberta Pancani, Aldo Paolicchi, Francesco Pistelli, Lorenzo Python, Massimo Santini, Laura Tavanti, Michele Tonerini, Federica Volpi, Chiara Zini.
